# Comparative study of self-expanding metal stent and intraluminal radioactive stent for inoperable esophageal squamous cell carcinoma

**DOI:** 10.1186/s12957-016-0768-x

**Published:** 2016-01-22

**Authors:** Dong Tian, Hongying Wen, Maoyong Fu

**Affiliations:** Cardiothoracic Surgery Department, Affiliated Hospital of North Sichuan Medical College, Nanchong, 637000 China

**Keywords:** Self-expanding metal stent, Intraluminal radioactive stent, Esophageal squamous cell carcinoma, Malignant esophageal stricture, Clinical curative effect

## Abstract

**Background:**

We compared the effectiveness of self-expanding metal stent alone vs. radioactive stent embedded with ^125^I seeds implantation insertion in patients of inoperable esophageal squamous cell cancer combined with malignant esophageal stenosis.

**Methods:**

We studied two groups of patients with stenosis attribute to inoperable esophageal squamous cell carcinoma. Group A had placed self-expanding metal stent alone insertion; group B encountered radioactive stent embedded with ^125^I seeds. Patients were followed up by monthly home visits or telephone interview. Survival time was analyzed with Kaplan-Meier analysis. Log rank test was used to analyze factors of survival time for all significant differences.

**Results:**

There was no significant difference between the two groups of all baseline characteristics. There was no statistical difference in complications including massive hematemesis, pain more than 1 month, stent migration, and restenosis. Survival time and causes of death such as tumor metastasis, massive hemorrhage, non-tumor-related factors, and restenosis were comparable between the two groups (*P* > 0.05). The medical costs were significantly less in group A than those in group B (*P* < 0.01).

**Conclusions:**

Radioactive stent embedded with ^125^I seeds was not significant in improving survival rate, but showed to increase hospitalization costs compared to self-expandable metal stent alone in treating inoperable esophageal squamous cell carcinoma stricture.

## Background

Esophageal cancer has a high morbidity and mortality rate than other malignant tumors. Dysphagia is the most common symptom in esophageal cancer, has serious impact on nutrient intake, and threatens the lives of patients [1, 2]. More than half of the patients need palliative treatment to relieve progressive esophageal stricture [3]. To remove the stenosis of esophageal cancer and improve the quality of life of patients with esophageal cancer, stent placement is the best choice to improve the symptom caused by dysphagia [4–7]. In 1983, Frimberger [8] treated a patient diagnosed with esophageal stricture placed with a metal stent. Metal stents became more and more popular for patients who encountered dysphagia [9, 10]. Recently, an esophageal stent embedded with iodine-125 (^125^I) seeds (also called intraluminal radiotherapy) has been developed [11, 12]. These two kinds of stents are more and more useful for inoperable esophageal cancer [13–17]. Both stents have been demonstrated as effective with few complications, although their relative effectiveness is unknown. However, there was great controversy to self-expanding metal stent and intraluminal radioactive stent in clinical curative effects, survival time, complications, hospital stay, and cost [11, 12, 18–24]. In our study, the self-expanding metal stent was compared with the radioactive stent embedded with ^125^I seeds in treating inoperable esophageal squamous cell carcinoma.

## Methods

### Patients

The study was approved by the institutional ethics committee of the Affiliated Hospital of North Sichuan Medical College. Informed consent was obtained from all enrolled patients. A total 131 patients with inoperable esophageal squamous carcinoma were selected for prospective non-randomized study. Group assignment was based on physician’s professional assessment as well as the patient’s own preference. Self-expandable metallic stent was performed in 91 patients (group A) and intraluminal radioactive stent was performed in 40 patients (group B) at the Affiliated Hospital of North Sichuan Medical College between September 2013 and June 2015.

The specific inclusion criteria were as follows:

1. The pathological diagnosis was esophageal squamous cell carcinomas.
2. Stenosis by tumor invasion.
3. Physical fitness score (Karnofsky score) ≥ 50
4. Expected to survive no less than 1 month.
5. No other organs encountered serious dysfunction.
6. No chemotherapy or radiotherapy was performed before and after stent implantation.
7. No chance to R0 resection for surgery (e.g., local lymphadenomegaly; tumor had invaded adjacent organ(s)).

The exclusion criteria were as follows:

1. Esophageal fistula.
2. The neck segment esophageal cancer.
3. Inability to obey follow-up.
4. Whatever the cause, stent placement was given up halfway.
5. Esophageal adenocarcinoma and esophagogastric junction carcinoma.
6. Patients rejected stent placement by personal reasons.

### Dysphagia score

Ogilvie’s dysphagia score classification is as follows: grade 0, to be able to eat the ordinary diet; grade 1, to be able to eat the solid food; grade 2, to be able to eat the semisolids; grade 3, to be able to take liquids food; and grade 4, to be able to eat nothing [25].

### Stent and ^125^I seeds

The system of self-expanding metal stent contains two elements: esophageal stent formed by nitinol and covered by silicone membrane. Besides the two parts, intraluminal radioactive stent is also composed of another nitinol sheath outside of the stent, which can contain ^125^I seeds (Nanjing Micro-tech Co., Ltd, Nanjing, China).

The radioactive seeds of ^125^I (Bejing ZHIBO Bio-medical Technology Co., Ltd, Beijing, China) are wrapped up in titanium alloy. Parameters of seeds are as follows: 4.5 mm, 0.8 mm, 60.1 days, 27–35 keV, 1.7 cm in length, diameter, half-life, mean photon energy, and tissue penetration. The original dosage rate was 7 cGy/h. Activity of each seed for clinical use is 0.80–0.90 mCi.

### Stent placement

Before stent placement, esophagoscope, computerized tomography (CT), and barium meal was used to confirm the tumor site, stricture degree, and length of tumor. The parameters of the stent were assessed according to the measured length of the tumor. The stent should be chosen more than 2 cm at both ends of the tumor. The stent implantation process of the two types of stents was in the same method. The esophagus was able to selectively enlarge according to the degree of the esophageal stenosis. Dilation was necessary when it was required to place the stent to a maximum of 11 mm by a Savary-Miller esophageal dilator (Changzhou Jiuhong Medical Instrument Co., Ltd.).

### Observation

Baseline characteristics were gender, age, tumor location, complications before stent placement, local lymph node enlargement (short diameter of lymph node ≥ 8 mm in the cervical region, mediastinum, abdomen), tumor stage (seventh edition of the AJCC and UICC), time of stent placement, length of tumor, length of stent, and esophagus dilation. Clinical curative effects were based on dysphagia score and tumor regression (smaller on the maximum section area of CT). The complications were sudden massive hemorrhage within the first month, pain more than 1 month, stent migration, and restenosis. Hospital stays and medical costs, survival time, and death causes were also identified.

### Followed up

Patients were followed up by monthly telephone interview or home visits from a specialized doctor. All the patients were followed up until July 2015 or death.

### Statistical analysis

Statistical software package SPSS 22.0 (SPSS, Inc., Chicago, IL) was used for all the data analyses. Mean ± standard deviation and range expressed the numeric data. Student’s *t* test, *χ* ^2^ test, Kaplan-Meier method, and log-rank test were applied in our study. *P* < 0.05 was considered to indicate a statistically significant difference.

## Results

### Patient characteristics

The median follow-up period in both groups A and B were 4 months. Stent placement was successful in both groups. The baseline characteristics of both groups were detailed in Table 1. There were no statistically significant differences in the gender, age, tumor location, complications before stent placement, local lymph node enlargement, tumor stage, time of stent placement, length of tumor, length of stent, and esophagus dilation between the two groups (*P* > 0.05).

**Table 1:** The baseline characteristics of studied groups

Characteristics	Group A (*n* = 91)	Group B (*n* = 40)	*P*
Gender, *n* (%)			0.869^c^
Male	67 (73.6 %)	30 (75 %)	
Female	24 (26.4 %)	10 (25 %)	
Age (years, mean ± SD) (range)	66.3 ± 9.4 (43–90)	66.9 ± 8.6 (47–84)	0.700^d^
Location of cancer, *n* (%)			0.195^c^
Proximal third	15 (16.5 %)	3 (7.5 %)	
Middle third	66 (72.5 %)	29 (72.5 %)	
Distal third	10 (11.0 %)	8 (20.0 %)	
Complications before stent placement, *n* (%)			0.601^c^
Yes	41 (46.1 %)	20 (50 %)	
No	50 (55.9 %)	20 (50 %)	
Local lymph node enlargement, *n* (%)^a^			0.686^c^
Yes	69 (75.8 %)	29 (72.5 %)	
No	22 (24.2 %)	11 (27.5 %)	
Tumor stage, *n* (%)^b^			0.437^c^
III	61 (67.0 %)	24 (60.0 %)	
IV	30 (33.0 %)	16 (40.0 %)	
Time of stent placement (min, mean ± SD) (range)	22.6 ± 4.9 (15–35)	20.2 ± 4.7 (14–32)	0.792^d^
Length of tumor (cm, mean ± SD) (range)	5.3 ± 1.3 (3–10)	5.5 ± 1.5 (2.5–8)	0.139^d^
Length of stent (cm, mean ± SD) (range)	9.3 ± 2.0 (6–16)	10.5 ± 1.6 (6–14)	0.274^d^
Esophagus dilation, *n* (%)			0.775^c^
Yes	57 (62.6 %)	24 (60 %)	
No	34 (37.4 %)	16 (40 %)	

^a^Short diameter of lymph node ≥8 mm in cervical region, mediastinum, abdomen

^b^7th edition of the AJCC&UICC

^c^ *χ* ^2^ exact test was used

^d^Student’s *t* test was used

### Clinical curative effects

The dysphagia score was improved in the two groups after stent placement. Three cases (7.5 %) of tumor growth were inhibited in group B after 3 months, group A did not, as shown in Table 2.

**Table 2:** Clinical curative effects

	Group A (*n* = 85)	Group B (*n* = 32)	*P*
Ogilvie’s dysphagia score (prior stent placement), *n* (%)			0.997^c^
3	66 (72.5 %)	29 (72.5 %)	
4	25 (27.5 %)	11 (27.5 %)	
Ogilvie’s dysphagia score (post stent placement)^a^, *n* (%)			0.200^c^
0	73 (80.2 %)	28 (70.0 %)	
1	18 (19.8 %)	12 (30.0 %)	
Tumor regression, *n* (%)^b^			0.008^c,d^
Yes	0 (0 %)	3 (7.5 %)	
No	91 (100 %)	37 (92.5 %)	

^a^ 3 days after stent implantation

^b^ Smaller on the maximum section area of CT

^c^ *χ* ^2^ exact test was used

^d^ *P* < 0.05 was considered to indicate a statistically significant difference

### Complications

There were no statistically significant differences in the sudden massive hemorrhage, pain more than 1 month, stent migration, or restenosis in both groups (*P* > 0.05), as shown in Table 3.

**Table 3:** Complications post to stent placement

	Group A, *n* (%)	Group B, *n* (%)	*P*
Massive hemorrhage^a^			0.337^b^
Yes	6 (6.6 %)	1 (2.5 %)	
No	85 (93.4 %)	39 (97.5 %)	
Pain more than 1 month			0.110^b^
Yes	16 (17.6 %)	12 (30.0 %)	
No	75 (82.4 %)	28 (70.0 %)	
Stent migration			0.908^b^
Yes	5 (5.5 %)	2 (5.0 %)	
No	86 (94.5 %)	38 (95.0 %)	
Restenosis			0.879^b^
Yes	4 (4.4 %)	2 (5.0 %)	
No	87 (95.6 %)	38 (95.0 %)	

^a^In the first month after stent placement

^b^ *χ* ^2^ exact test was used

(1) Massive hemorrhage: No patient encountered sudden massive hemorrhage during stent placement, but this complication developed in six (6.6 %) and one (2.5 %) patients in group A and group B, respectively, post placement; all of which were fatal.

(2) Pain: Most of the patients encountered retrosternal pain after the stent placement and part of the patients felt severe chest pain, which was relieved using narcotic analgesics. Sixteen patients in group A and 12 patients in group B felt severe pain more than 1 month, and no statistically significant differences were identified between the two groups (*P* > 0.05).

(3) Stent migration: Five patients in group A and two patients in group B experienced stent migration 2 months after stent placement, confirmed by electronic endoscopy or barium meal. Re-intervention by gastroscope assistance was successful on these seven patients.

(4) Restenosis: Four patients in group A and two patients in group B experienced restenosis 3 months after stent placement, confirmed by barium meal and/or gastroendoscopy. No patient chose to undergo further treatment.

(5) Others: There were no radioactive pneumonia, bone marrow suppression, gastrointestinal reaction, and other complications related to radiotherapy in group B.

### Hospital stays and medical costs

The hospital stays were shorter in group A than those in group B, but this did not reach statistical significance difference: 8.9 vs. 10.1 days, respectively (*P* > 0.05). The medical costs were significantly less in group A than those in group B: ¥7000 vs. ¥26,000, respectively (*P* < 0.01).

### Survival time and death causes

**Fig. 1:**
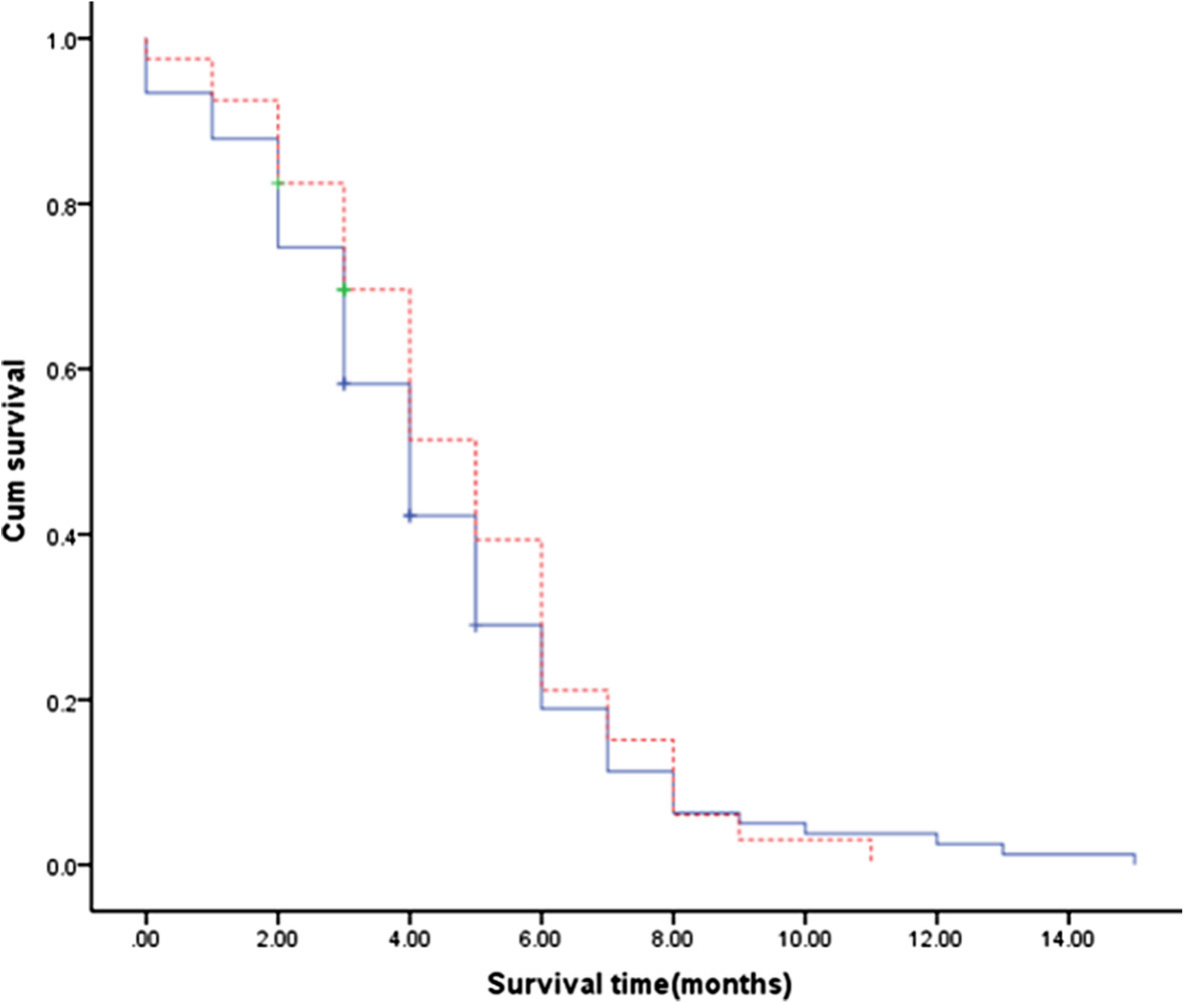
Intraluminal radioactive stent self-expanding metal stent. Kaplan-Meier curves of overall survival. Comparison of overall survival between group A (self-expanding metal stent) and group B (intraluminal radioactive stent). The mean overall survival period was similar in group A compared with that in group B: 4.2 ± 2.8 (0–15) months vs. 4.4 ± 2.4 (0–9) months, respectively. There was no significant difference in survival time (*P* > 0.05)

**Fig. 2:**
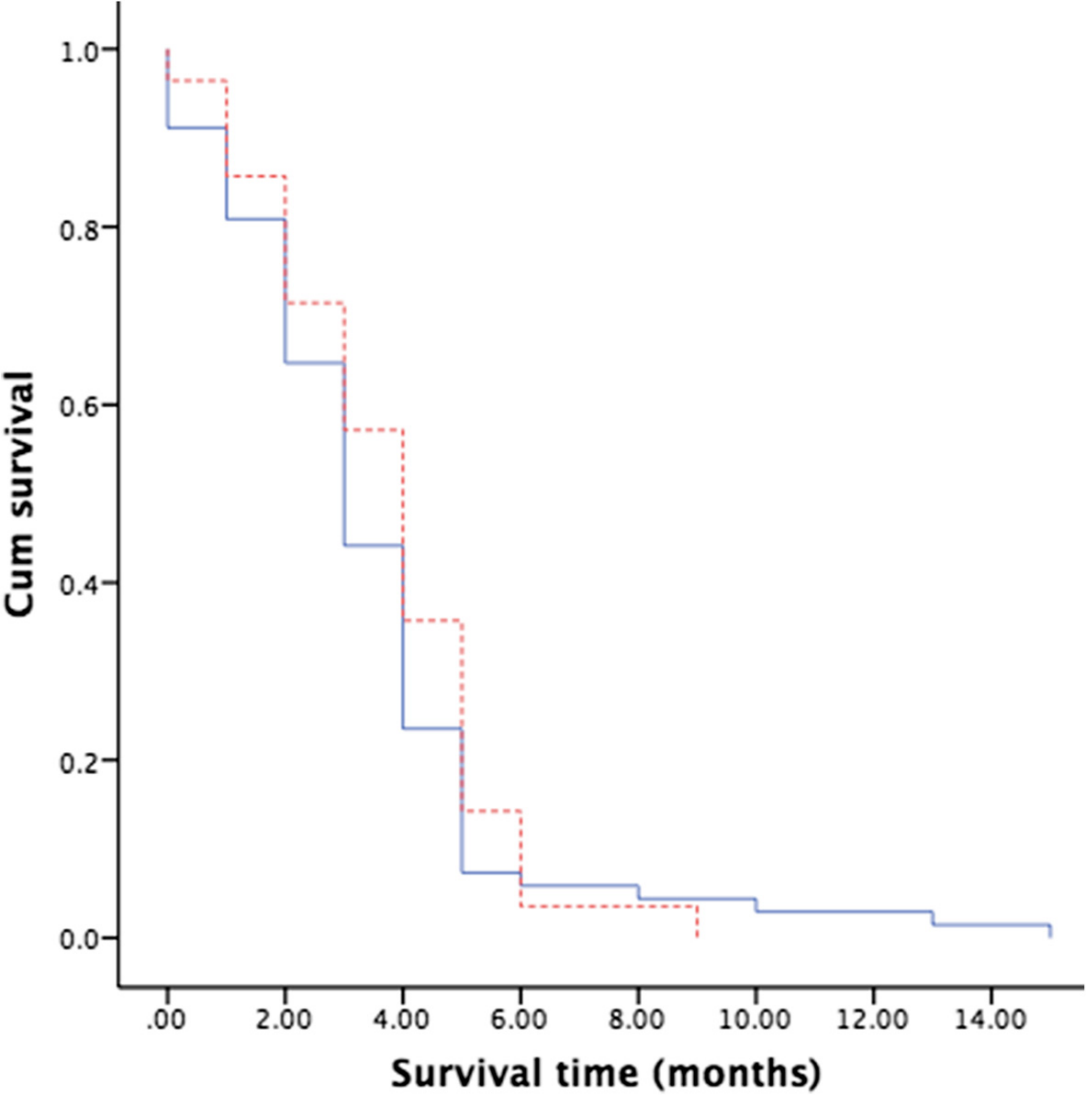
Intraluminal radioactive stent, self-expanding metal stent. Kaplan-Meier curves of disease-specific survival. Comparison of disease-specific survival between group A (self-expanding metal stent) and group B (intraluminal radioactive stent). The mean disease-specific survival period was similar in group A compared with that in group B: 3.4 ± 0.3 months vs. 3.7 ± 0.4 months, respectively. There was no significant difference in survival (*P* > 0.05)

The mean survival time in group A and group B was 4.2 ± 2.8 (0–15) months and 4.4 ± 2.4 (0–9) months, respectively. The Kaplan-Meier curves were shown in Fig. 1. There was no significant differences in the survival time (*P* = 0.752 > 0.05). The main death causes were tumor metastasis (40.7 % vs. 40.0 %) and massive hemorrhage (30.8 % vs. 27.5 %), as shown in Table 4. Among these death causes, 96 patients (group A = 68, group B = 28) died of esophageal cancer. The mean disease specific survival time in group A and group B was 3.4 ± 0.3 and 3.7 ± 0.4 months, respectively. The Kaplan-Meier curves were shown in Fig. 2. There was no significant difference in survival (*P* = 0.421 > 0.05).

**Table 4:** Death causes

Death causes	Group A (*n* = 86)	Group B (*n* = 35)	*P =* 0.959
Tumor metastasis, *n* (%)	37 (40.7 %)	16 (40.0 %)	
Massive hemorrhage, *n* (%)	28 (30.8 %)	11 (27.5 %)	
Non-tumor-related factors, *n* (%)	18 (19.8 %)	7 (17.5 %)	
Restenosis, *n* (%)	3 (1.1 %)	1 (2.5 %)	

*χ* ^2^ exact test was used

## Discussion

Advanced esophageal squamous cell carcinoma may cause dysphagia. Dysphagia is the main symptom of patients with inoperable esophageal squamous cell carcinoma [1]. Most of them need the palliative care to relieve this symptom [3].

Although esophageal bypass surgery has been performed to enable oral ingestion, the outcome of this palliative surgery is not always favorable because it is extremely invasive for the patients in an unstable condition as a result of advanced cancer [26–28]. In contrast, the insertion of an esophageal stent has many advantages for such patients because it is much less invasive and the procedure is simpler and safer than bypass surgery. Recently, stenting has been considered to be the best way of treatment for malignant stenosis.

These work compared the self-expanding metal stent alone and intraluminal radioactive stent loaded with ^125^I seeds for palliation of dysphagia due to inoperable squamous carcinoma of the esophagus.

Although various studies have described, intraluminal radioactive stent got a better long-term clinical effect of dysphagia and went with fewer complications. Moreover, the median survival time was also longer in intraluminal radioactive stent than self-expanding metal stent alone. Our study showed no significance evidence of this fact in inoperable esophageal squamous cell carcinoma.

All patients in our study were inoperable of advanced esophageal cancer who chose self-expanding metal stent (group A) and the radioactive stent embedded with ^125^I seeds (group B) voluntarily. Both stent placements were clinically successful. There was no death during stent implantation.

The score of dysphagia was improved greatly in all patients after stenting, and there was no statistically significant difference between groups A and group B. Three months after stent placement, 4 patients in group A and 2 patients in group B experienced restenosis. The causes of restenosis after placement may be due to the granulation hyperplasia tissue and/or tumor tissue growing into the ends of the stent [24]. No patient in our study encountered restenosis because of the regrowth of the tumor tissue.

Three months later, three cases (7.5 %) with tumor growth was inhibited in group B, group A did not. This demonstrated that the intraluminal radioactive stent had the effect of inhibiting tumor growth and the hyperplasia of the granulation tissue.

The main complications following the stent placement include massive hematemesis, pain more than 1 month, stent migration, and restenosis [13, 29–33]. There was no statistical significance difference between group A and group B in these outcomes.

Hemorrhaging after stent placement has been reported about 3–8 % and most of the time was self-limited [1]. This incidence has also been reported as much as 30 % in part of the study [11]. In our study, six patients in group A and one in group B encountered massive hemorrhaging in 1 month after stent placement, but no patients succumbed during the process of stent placement.

In our study, most of the patients encountered retrosternal pain after the stent placement and part of the patients felt severe chest pain, which was relieved using narcotic analgesics. Sixteen patients in group A and 12 patients in group B felt severe pain more than 1 month, and no statistically significant difference was identified between the two groups. That was lower than has been observed in previous research (53.8 %) [34]. No patient stopped stent treatment because of severe pain.

Five cases in group A and two cases in group B experienced stent migration 2 months after stent placement, confirmed by electronic endoscopy or barium meal. Reintervention by gastroscope assistance was successful on these patients. That should be due to the nature of the nickel-titanium shape memory alloy stents. Cold temperature will make the stent soften. When the temperature reaches more than 37 °C for 1 week, the stent will form a shape that does not change in response to cold temperatures. In order to prevent the stent from deformation or shifting, cold foods could be taken no less than 1 week after stenting [24].

The hospital stays were shorter in group A than those in group B, but there was no statistically significant differences: 8.9 vs. 10.1 days, respectively. Few studies reported hospital stays, but found longer compared to those of our study. The medical costs were significantly less in group A than those in group B: ¥7000 vs. ¥26,000, respectively. Some of other previous studies found that there was no significant differences between the total medical costs of metal stent placement vs. radiotherapy in the palliative treatment of esophageal squamous cell cancer [18, 19].

The mean survival time in group A and group B was 4.2 ± 2.8 (0–15) months and 4.4 ± 2.4 (0–9) months, respectively. There was no statistical significance difference in survival time (*P* > 0.05). Besides, the disease-specific survival better reflects the impact of stent placement on the survival of patients. In this study, the mean disease-specific survival time in group A and group B was 3.4 ± 0.3 and 3.7 ± 0.4 months, respectively. There was also no statistical significance difference in survival (*P* > 0.05). The main death causes were tumor metastasis (40.7 % vs. 40.0 %) and massive hemorrhage (30.8 % vs. 27.5 %). Although various studies [11, 12, 18, 35] have described a longer survival time of intraluminal radioactive stent than that of self-expanding metal stent, our results showed no evidence of significance supporting this fact in inoperable esophageal squamous cell cancer. The reason of this difference may be attributed to different case compositions. All the patients in our research were only at advanced stage (III, IV stage) and esophageal squamous cell carcinoma; however, other studies included early/advanced stage (II, III, IV stage) patients and esophageal adenocarcinoma/adenocarcinoma of the esophagogastric junction.

Theoretically, radioactive stent embedded with ^125^I seeds implantation may be better than self-expanding metal stent alone insertion because of the different types of stent to the tumor. Intraluminal radioactive stent provides closer, longer continuous radiotherapy with tumors. Nevertheless, the dose of radiation with radioactive stent in the esophageal lumen was too difficult to precisely measure and plan. Therefore, an accurate dose of ^125^I seeds was important. Unfortunately, there was no treatment planning system (TPS) for luminal organs. The TPS that we used mainly depended on the design of solid tumors.

In the present study, the main death causes were tumor metastasis and massive hemorrhage. Although three cases of tumor growth were inhibited, there was no significant effect of radioactive stent to inhibit tumor metastasis and massive hemorrhage.

Some inevitable limitations also could be found in our study. Firstly, the sample size of the present study was considered small, although it reached the statistical significance which could be due to the time limitation and inclusion criteria. Our further studies with bigger sample sizes may make more accurate results. Secondly, the quality of life for these patients, an important measure to assess for the palliative treatment of cancer, including inoperable esophageal squamous cell cancer, was not measured in our study.

## Conclusions

Intraluminal radioactive stent embedded with ^125^I seeds implantation was not significant when it came to the outcome of survival benefit, but increased the hospitalization expense than self-expandable metal stent alone in treating inoperable esophageal squamous cell carcinoma stricture.

## Consent for publication

Informed consent for publishing the individual patient data was obtained from all the participants.
